# Preclinical evaluation of combination nemtabrutinib and venetoclax in chronic lymphocytic leukemia

**DOI:** 10.1186/s13045-022-01386-1

**Published:** 2022-11-15

**Authors:** Elizabeth M. Muhowski, Janani Ravikrishnan, Britten Gordon, Lianbo Yu, Shrilekha Misra, Brandi Walker, Sudharshan Eathiraj, Deepa Sampath, Kerry A. Rogers, John C. Byrd, Jennifer A. Woyach

**Affiliations:** 1 Division of Pharmaceutics and Pharmacology, College of Pharmacy, The Ohio State University, Columbus, OH USA; 2 Division of Hematology, Department of Internal Medicine, The Ohio State University, 410 W 12Th Avenue, Columbus, OH 43210 USA; 3 Center for Biostatistics, Department of Biomedical Informatics, The Ohio State University, Columbus, OH USA; 4 ArQule, a Wholly-Owned Subsidiary of Merck & Co., Inc., Kenilworth, NJ USA; 5 Division of Hematopoietic Biology and Malignancy, MD Anderson Cancer Center, Houston, TX USA; 6 Department of Internal Medicine, University of Cincinnati, Cincinnati, OH USA

**Keywords:** CLL, Nemtabrutinib, Ibrutinib, Venetoclax

## Abstract

Inhibitors of B cell receptor (BCR) signaling such as the Bruton’s tyrosine kinase (BTK) inhibitors are effective therapeutics for chronic lymphocytic leukemia (CLL). The first-in-class covalent BTK inhibitor, ibrutinib, produces durable responses in most CLL patients; however, complete responses are only observed in a minority of patients. B cell lymphoma 2 (BCL2), an anti-apoptotic protein that contributes to CLL cell survival, has also been investigated as a therapeutic target. The BCL2 inhibitor venetoclax is effective in patients with CLL and can produce undetectable minimal residual disease, allowing discontinuation of therapy. In combination, ibrutinib and venetoclax have shown preclinical synergy and clinical efficacy. Nemtabrutinib is a next generation, reversible inhibitor of BTK that potently inhibits BCR signaling in treatment-naïve and ibrutinib-refractory CLL cells ex vivo. The clinical efficacy of combining BTK inhibitors with BCL2 inhibitors motivated us to evaluate the novel combination of nemtabrutinib and venetoclax. In vitro studies show that nemtabrutinib and venetoclax are not antagonistic to each other. In an adoptive transfer CLL mouse model, mice treated with nemtabrutinib and venetoclax had prolonged survival compared to mice treated with ibrutinib and venetoclax. Our preclinical studies further validate the combination of BTK inhibitors with venetoclax and justify further investigation of combining nemtabrutinib with venetoclax in CLL.

**Supplementary Information:**

The online version contains supplementary material available at 10.1186/s13045-022-01386-1.

**To the editor**

Many antagonists of B cell receptor (BCR) signaling, especially inhibitors of Bruton’s tyrosine kinase (BTK), have demonstrated clinical efficacy in the treatment of chronic lymphocytic leukemia (CLL). While therapeutically effective, BTK inhibition alone is not sufficient to eliminate disease, with only a small subset of patients treated with the BTK inhibitor (BTKi) ibrutinib reaching complete responses and rarely achieving undetectable minimal residual disease (uMRD) [1]. Previous studies showed prolonged BTK inhibition increases dependence on B Cell Lymphoma 2 (BCL2), an anti-apoptotic protein highly expressed in CLL [2]. In a phase 1 clinical trial, patients with relapsed/refractory (R/R) CLL treated with the BCL2 inhibitor venetoclax had an overall response rate of 79%; however, only 5% of patients had uMRD in the bone marrow [3]. The combination of ibrutinib and venetoclax is preclinically synergistic and clinically effective [2, 4, 5]. Nemtabrutinib (formerly ARQ 531, MK-1026) is a potent, reversible BTKi that has demonstrated greater inhibition of BTK’s downstream targets compared to ibrutinib, as well as improved efficacy in R/R disease and Richter’s Transformation [6]. Therefore, we aimed to determine if combination nemtabrutinib and venetoclax would be similar or superior to combination ibrutinib and venetoclax in preclinical testing.

We observed significantly decreased viability of primary CLL cells treated with venetoclax compared to vehicle (*p* < 0.0001); though not statistically significant, the addition of ibrutinib or nemtabrutinib further increased cytotoxicity by 6 and 10%, respectively, compared to venetoclax alone (Fig. 1A). In primary CLL cells with C481S BTK, venetoclax significantly decreased viability compared to vehicle (*p* = 0.0074). While not statistically significant due to small sample size, nemtabrutinib decreased cell viability and ibrutinib was ineffective (Fig. 1B). Next, we investigated the effects of combining venetoclax and BTKis on BCR signaling (Fig. 1C). Both BTKis inhibited autophosphorylation of BTK^Y223^ (ibrutinib vs. stimulated vehicle *p* = 0.0021, nemtabrutinib vs. stimulated vehicle *p* = 0.0024), which was unaffected by addition of venetoclax (Fig. 1D). As previously shown, ERK phosphorylation was inhibited to a greater degree with nemtabrutinib than ibrutinib (nemtabrutinib vs. stimulated vehicle *p* < 0.0001, nemtabrutinib vs. ibrutinib *p* = 0.0461), and, as expected, this inhibition was not affected by the addition of venetoclax (Fig. 1E) [6].

**Fig. 1:**
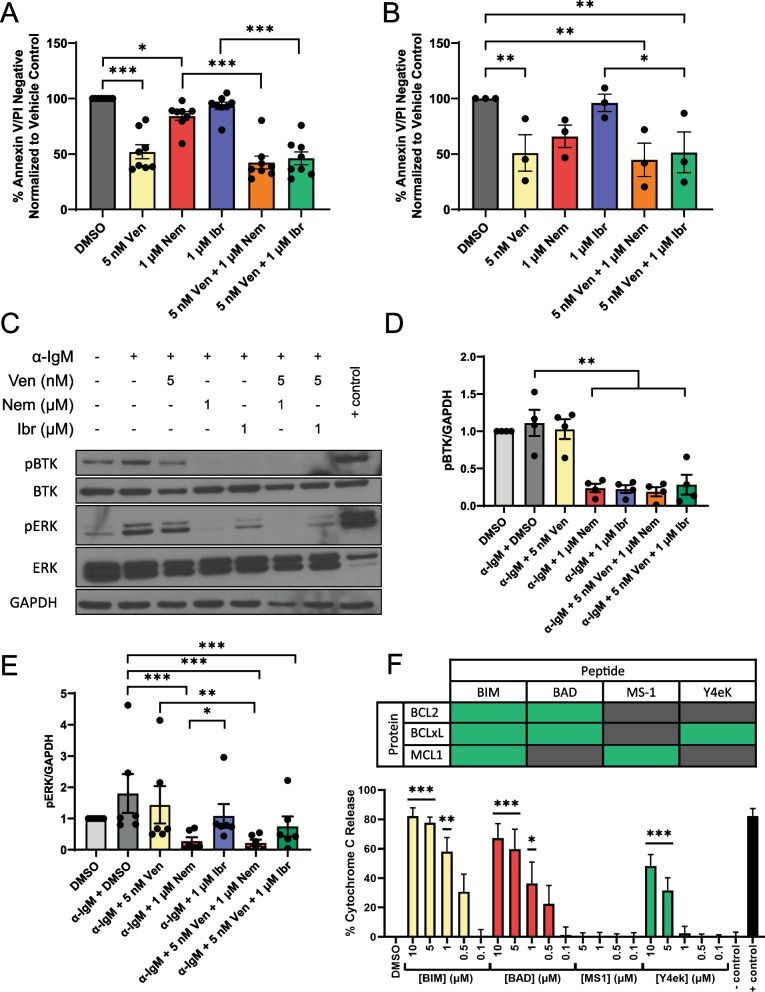
**A** Primary CLL cells were treated with DMSO, venetoclax (5 nM), nemtabrutinib (1 μM), ibrutinib (1 μM) or in combination. Ibrutinib was washed out after 1 h and total incubation time was 24 h. Cell viability was assessed by flow cytometry following Annexin V/PI staining. Data are normalized to DMSO control. A linear mixed effect model was used to analyze raw data and p values were adjusted for multiple comparisons using Tukey’s method (*p ≤ 0.05, **p ≤ 0.01, ***p ≤ 0.001). **B** Ibrutinib refractory CLL cells harboring C481S mutant BTK were treated with DMSO, venetoclax (5 nM), nemtabrutinib (1 μM), ibrutinib (1 μM) or in combination. Ibrutinib was washed out after 1 h and total incubation time was 24 h. Cell viability was assessed by flow cytometry following Annexin V/PI staining. Data are normalized to DMSO control. A linear mixed effect model was used to analyze raw data and p values were adjusted for multiple comparisons using Tukey’s method (*p ≤ 0.05, **p ≤ 0.01, ***p ≤ 0.001). **C** Representative immunoblot. Primary CLL cells were treated with DMSO, venetoclax (5 nM), nemtabrutinib (1 μM), ibrutinib (1 μM) or in combination. Ibrutinib was washed out after 1 h and total incubation time was 24 h. Cells were then stimulated with 10 µg plate-bound anti-IgM for the final 15 min of the total incubation time before collection of whole cell lysate and analysis using SDS-PAGE. **D, E** All immunoblots of primary CLL patient samples were quantified using densitometry software (AlphaView). Protein levels (**D.** pBTK, **E.** pERK,) normalized to GAPDH loading control are reported as fold change over vehicle control. A linear mixed effect model was used to analyze raw data normalized to GAPDH loading control and p values were adjusted for multiple comparisons using Tukey’s method (*p ≤ 0.05, **p ≤ 0.01, ***p ≤ 0.001). **F** Primary CLL cells from nemtabrutinib treated patients were co-cultured with NK Tert stromal cells (Riken, RCB2350) before performing BH3 profiling using BIM, BAD, MS-1, Y4ek, and PUMA2A peptides [8]. Baseline cytochrome c release measured in DMSO treated cells has been subtracted from all test conditions and controls presented. Cytochrome c release in response to interaction with BIM, BAD, and Y4ek peptides indicates cellular dependency on BCL2 and BCL-xL. A linear mixed effect model was used to analyze raw data and p values were adjusted for multiple comparisons using Tukey’s method. All statistics represented are in comparison to DMSO treated control (*p ≤ 0.05, **p ≤ 0.01, ***p ≤ 0.001). Peptide-protein interaction chart shows high interaction in green and low interaction in gray

We additionally sought to determine the dependence on BCL2-family proteins in CLL cells from patients treated with nemtabrutinib in a clinical trial at our institution. BH3 profiling of samples from 3 nemtabrutinib treated patients showed cytochrome c release in response to interaction with BIM, BAD, and Y4ek peptides, indicating that these CLL cells are dependent on BCL2 and BCL-xL (Fig. 1F) [7]. This suggests venetoclax sensitivity and supports the potential clinical benefit of combining nemtabrutinib and venetoclax.

Next, we tested the in vivo efficacy of venetoclax in combination with either BTKi using the Eμ-TCL1 adoptive transfer model (Fig. 2A) [9]. Due to the large study size, splenocytes from multiple Eμ-TCL1 donors were required. Peripheral blood disease was monitored weekly (Fig. 2B). As a result of using different donor clones, we observed high variability between recipient groups, as previously reported by Kater et al. [10] including groups that did not have BCR-dependent disease as evidenced by lack of response to ibrutinib. These groups were excluded from analysis, but data are presented in Additional file 1: Fig. S1. We assessed the survival of 48 mice engrafted with splenocytes from a single donor with BCR-dependent disease (Fig. 2C, primary statistical analysis in legend). Ibrutinib-treated mice reached a median survival of 56.5 days while mice treated with ibrutinib and venetoclax had a median survival of 66 days. Nemtabrutinib-treated mice reached a median survival of 81.5 days while mice treated with nemtabrutinib and venetoclax reached a median survival of 92 days. Interestingly, mice treated with venetoclax alone did not prolong survival compared to vehicle-treated mice. Mice treated with nemtabrutinib alone or in combination had significantly prolonged survival compared to vehicle-treated mice (*p* = 0.0238, *p* < 0.0001, respectively). Notably, the combination of nemtabrutinib and venetoclax significantly prolonged survival compared to the combination of ibrutinib and venetoclax (*p* = 0.0415).

**Fig. 2:**
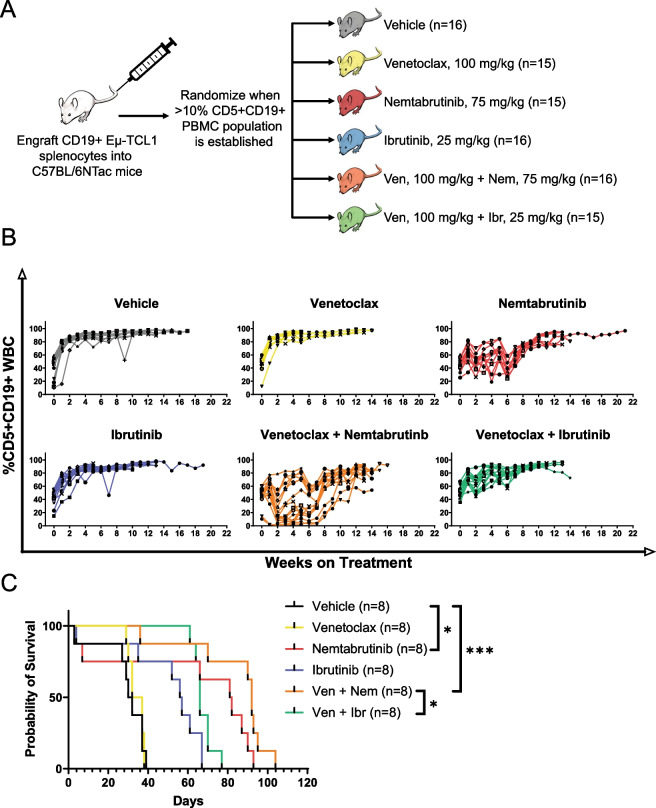
**A** Splenocytes from 4 Eμ-TCL1 donors were engrafted into C57BL/6NTac mice (*n* = 93) via tail vein injection in groups of 48, 25, 17, and 3 recipient mice, respectively. Recipient mice were randomly enrolled into treatment groups after developing CD5 + CD19 + disease as assessed by flow cytometry of peripheral blood. Mice were treated daily via oral gavage with vehicle, venetoclax (100 mg/kg), nemtabrutinib (75 mg/kg), ibrutinib (25 mg/kg), venetoclax (100 mg/kg) and nemtabrutinib (75 mg/kg), or venetoclax (100 mg/kg) and ibrutinib (25 mg/kg). Mice receiving single agents were administered two gavages: one gavage containing drug and one gavage containing vehicle. Mice receiving drug combinations were administered two gavages containing each drug separately. **B** Disease progression of all recipient mice (*n* = 93). Peripheral blood disease progression was monitored weekly via flow cytometry and is reported as %CD5 + CD19 + of CD45 + cells. **C** Kaplan–Meier estimated survival of recipient mice engrafted with splenocytes from a single Eµ-TCL1 donor with BCR-dependent disease (*n* = 48). A log rank test was used to assess the primary statistical comparisons (vehicle vs. nemtabrutinib, vehicle vs. nemtabrutinib and venetoclax, nemtabrutinib vs. nemtabrutinib and venetoclax, and nemtabrutinib and venetoclax vs. ibrutinib and venetoclax). Holm’s method was used to adjust *p* values to correct for multiple comparisons (*p ≤ 0.05, **p ≤ 0.01, ***p ≤ 0.001)

The data presented here further demonstrate the benefit of combining BTKis with venetoclax in CLL and suggest that nemtabrutinib is a rational BTKi to combine with venetoclax. Our in vivo data especially support the combination of nemtabrutinib plus venetoclax as an alternative to ibrutinib plus venetoclax. Further investigation, both preclinically and clinically, is warranted.

## Supplementary Information

**Additional file 1: Fig. S1.** Kaplan–Meier estimated survival of all recipient mice (n=93) treated with vehicle, venetoclax (100 mg/kg), nemtabrutinib (75 mg/kg), ibrutinib (25 mg/kg), venetoclax (100 mg/kg) and nemtabrutinib (75 mg/kg), or venetoclax (100 mg/kg) and ibrutinib (25 mg/kg).

## Abbreviations

BCR: B cell receptor
BTK: Bruton’s tyrosine kinase
BTKi: Bruton’s tyrosine kinase inhibitor
CLL: Chronic lymphocytic leukemia
uMRD: Undetectable minimal residual disease
BCL2: B cell lymphoma 2

## Declarations

### Availability of data and materials

The data that support the findings of this study are available on request from the corresponding author.
