# Supplementary figures and images for: Preclinical evaluation of combination nemtabrutinib and venetoclax in chronic lymphocytic leukemia
Source: J Hematol Oncol. 2022 Nov 15;15:166. doi: 10.1186/s13045-022-01386-1 (PMC9664697; doi:10.1186/s13045-022-01386-1)

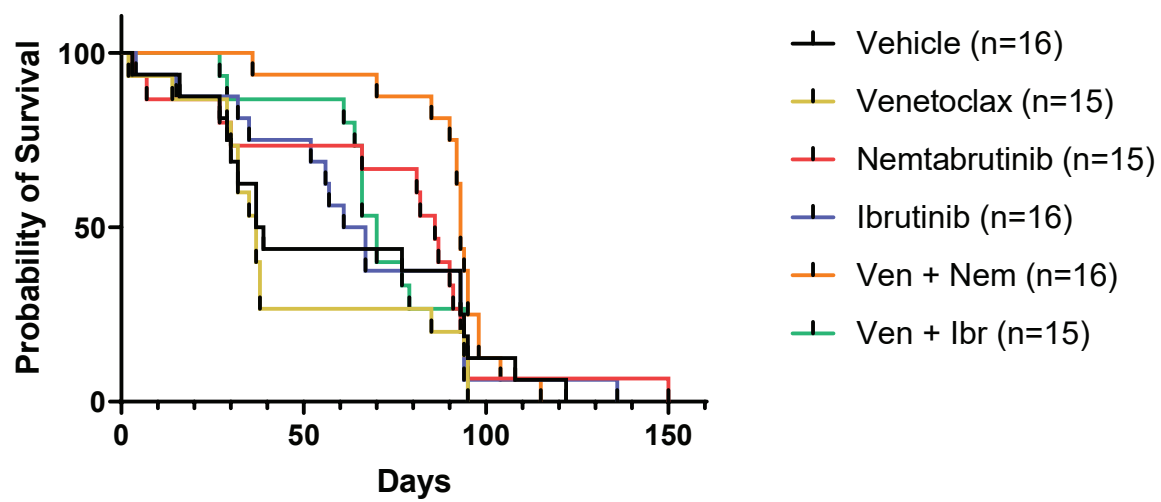

Supplement: Supplementary file 1 — Additional file 1: Fig. S1. Kaplan–Meier estimated survival of all recipient mice (n=93) treated with vehicle, venetoclax (100 mg/kg), nemtabrutinib (75 mg/kg), ibrutinib (25 mg/kg), venetoclax (100 mg/kg) and nemtabrutinib (75 mg/kg), or venetoclax (100 mg/kg) and ibrutinib (25 mg/kg). [file 13045_2022_1386_MOESM1_ESM.pdf]
